# Supplementary material for: Cryptosporidium sequentially remodels its single rhoptry into the host interface
Source: bioRxiv. 2026 Jul 21:2026.07.21.739819. Preprint. [Version 1] doi: 10.64898/2026.07.21.739819 (PMC13419397; doi:10.64898/2026.07.21.739819)
Supplement: 1 [file NIHPP2026.07.21.739819v1-supplement-1.pdf]

bioRxiv preprint doi: https://doi.org/10.64898/2026.07.21.739819; this version posted July 21, 2026. The copyright holder for this preprint (which was not certified by peer review) is the author/funder, who has granted bioRxiv a license to display the preprint in perpetuity. It is made available under a CC-BY 4.0 International license.

Supplementary figures and legends

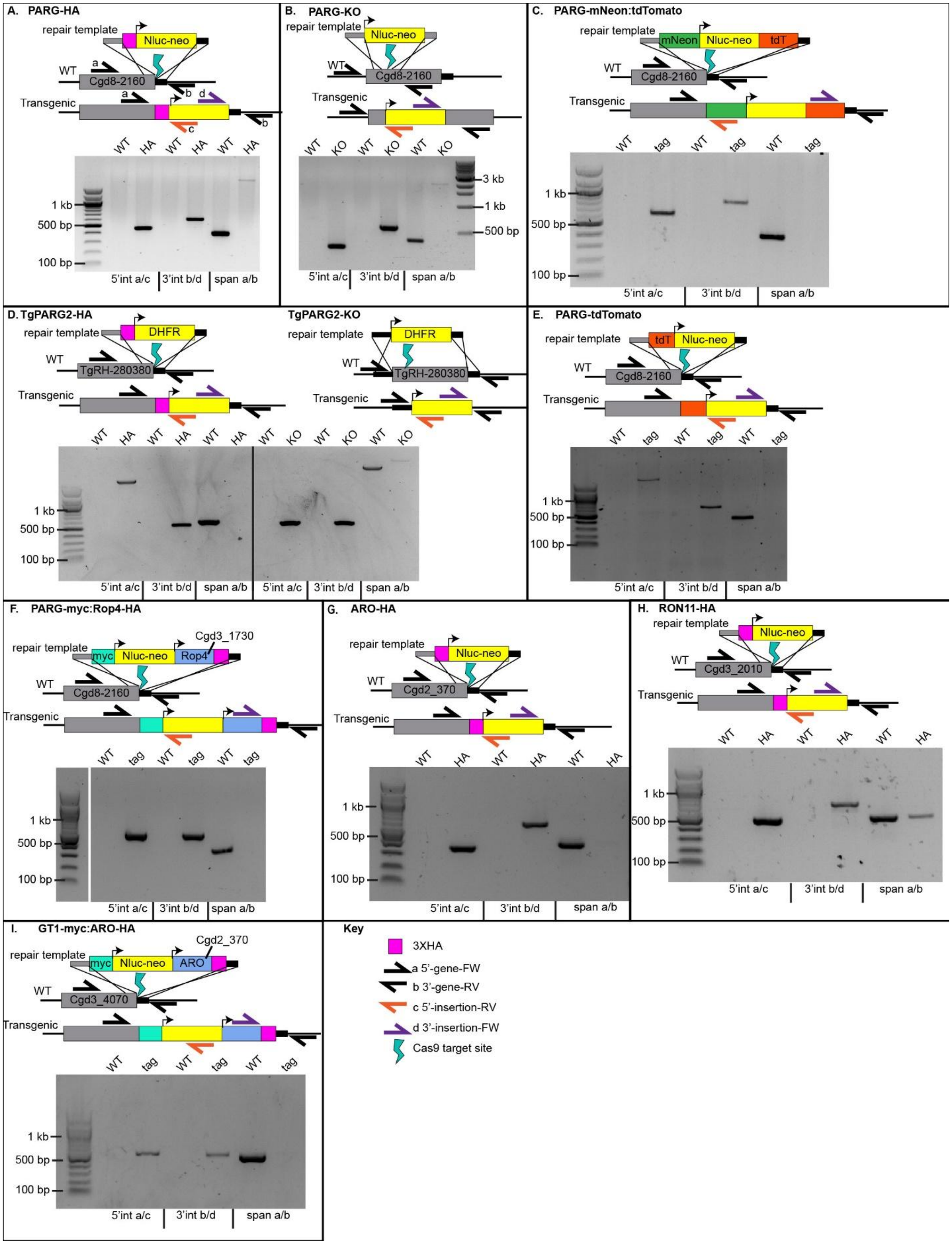

bioRxiv preprint doi: https://doi.org/10.64898/2026.07.21.739819; this version posted July 21, 2026. The copyright holder for this preprint (which was not certified by peer review) is the author/funder, who has granted bioRxiv a license to display the preprint in perpetuity. It is made available under a CC-BY 4.0 International license.

Figure S1. Strain generation. CRISR-CAS9 targeting and homology-directed repair design are shown in a schematic. Promoters are annotated as bent arrows, and primers for checking integration of the construct are annotated as split arrows. See Table S3 for more details on the primers used for integration PCR and the expected amplicon sizes for each transgenic. The gel corresponding to integration PCR for each transgenic is shown below the schematic in inverted contrast. The sample (WT or transgenic at the indicated locus) are labelled at the top of the lane, and the PCR/primers used are labelled at the bottom. Key ladder markers are annotated on the left or right of the image. Transgenic lines are as follows: **A.** PARG (Cgd8_2160) HA epitope tagging **B.** PARG-KO – nluc-neo is knocked into the 5' end of the ORF, excising half of the PARG domain and splitting the ORF **C.** PARG-mneon:tdTomato – PARG tagging with mNeon and cytosolic expression of tdTomato attached to nluc-neo via a self-cleaving T2A peptide. **D.** *T. gondii* dense granule PARG (280380, TgPARG2) HA epitope tagging and TgPARG2 knockout by gene replacement **E.** PARG-tdT – PARG tagging with tdTomato **F.** PARG-myc:ROP4-HA – PARG is myc-tagged at its endogenous locus and CpROP4-HA is knocked in downstream of the nluc:neo cassette with 500bp of sequence upstream of the start codon to include the native promoter **G.** ARO (Cgd2_370) HA epitope tagging **H.** RON11 (Cgd3_2010) epitope tagging. **I.** GT1 (Cgd3_4070) myc-tagging with ARO-HA (Cgd2_370) with 500bp of sequence upstream of the start codon is knocked in downstream of the nluc-neo cassette.

bioRxiv preprint doi: https://doi.org/10.64898/2026.07.21.739819; this version posted July 21, 2026. The copyright holder for this preprint (which was not certified by peer review) is the author/funder, who has granted bioRxiv a license to display the preprint in perpetuity. It is made available under a CC-BY 4.0 International license.

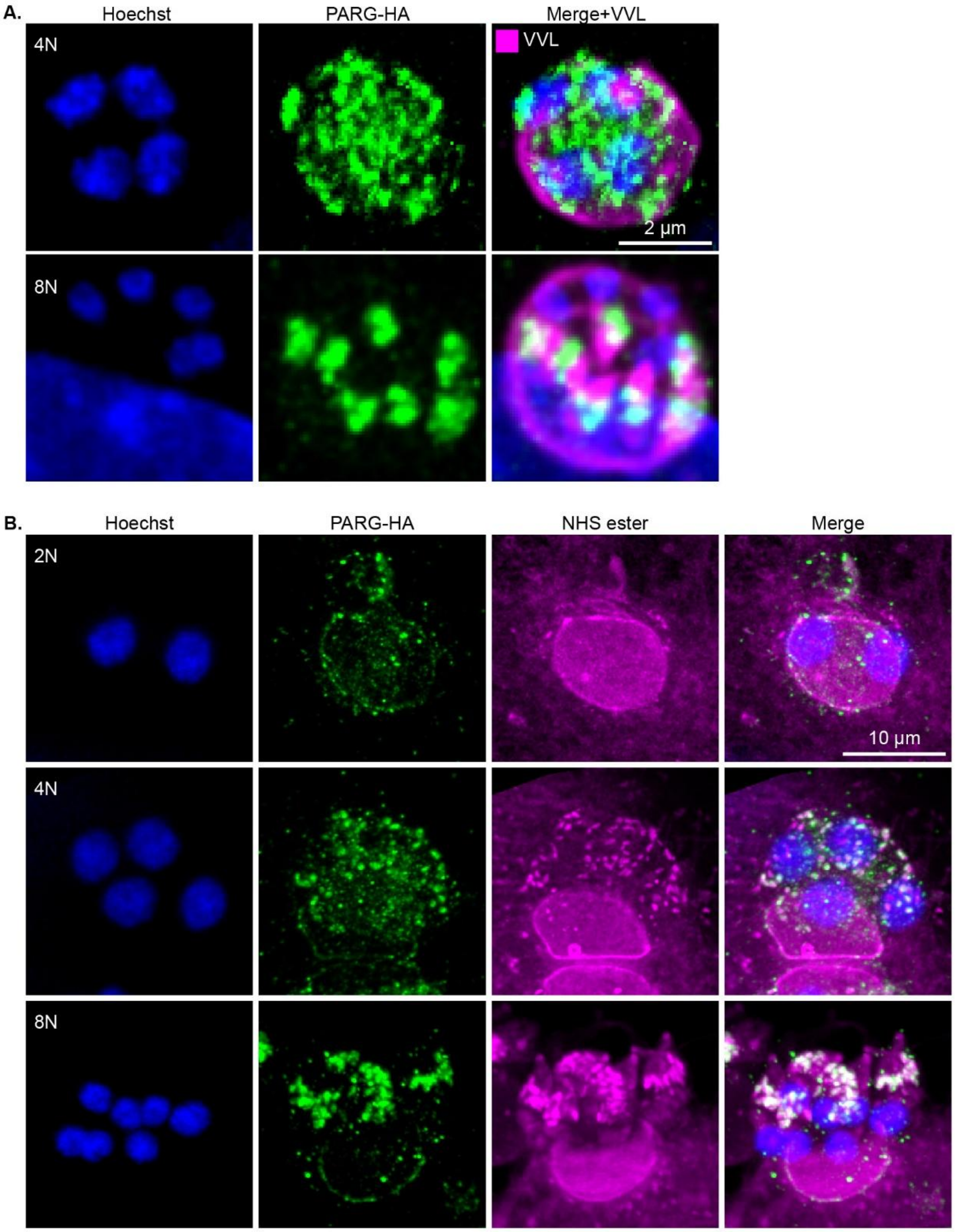

Figure S2. Dense granule synthesis occurs after 2 nuclear divisions. A. Immunofluorescence microscopy showing the distribution of PARG-HA in parasites undergoing asexual replication in HCT-8 cells. Newly synthesized PARG is detected in intracellular parasites starting after 2 nuclear divisions (4N). At the third division, when the parasites have 8 nuclei, the PARG-HA signal coalesces into discrete puncta located anterior to the nucleus of the budding merozoites. **B.** Expansion microscopy of parasites undergoing asexual replication showing PARG-HA staining, protein density, and number of nuclei in 2N, 4N, and 8N meronts. Increased PARG-HA expression

bioRxiv preprint doi: https://doi.org/10.64898/2026.07.21.739819; this version posted July 21, 2026. The copyright holder for this preprint (which was not certified by peer review) is the author/funder, who has granted bioRxiv a license to display the preprint in perpetuity. It is made available under a CC-BY 4.0 International license.

was observed in 4N meronts. The individual PARG-HA-positive puncta were smaller and more numerous in 4N than in 8N parasites. The intensity of PARG-HA staining at the dense band did not increase noticeably during merogony.

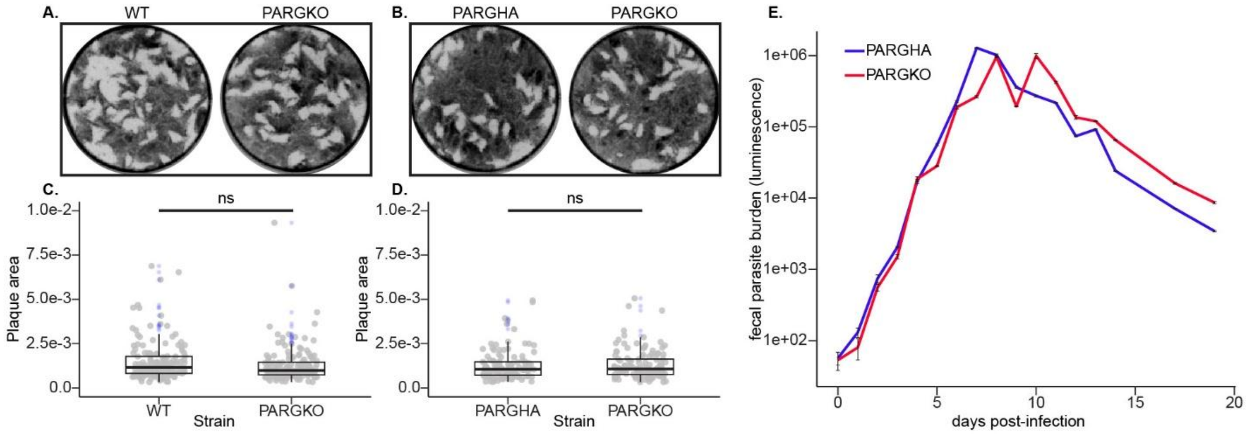

Figure S3. Dense granule PARG-domain containing proteins are not required for parasite fitness. A-B. HFF monolayers were infected with serial dilutions of the indicated *T. gondii*. Monolayers were fixed, stained with Giemsa, and imaged one week after infection. **C-D.** Plaque areas of three independent biological replicates per condition were segmented and their area measured. There was no significant difference in plaque area when comparing WT/TgPARG2-HA and TgPARG2-KO *T. gondii* strains. **E.** *Ifnγ*$^{-/-}$ mice were infected with passage-matched PARG-HA and PARG-KO *C. parvum* oocysts. Fecal luminescence was measured to assess parasite shedding over time. No difference in parasite shedding was observed in PARG-KO compared to PARG-HA *C. parvum.* Representative fecal luminescence curve from 3 independent experiments.

bioRxiv preprint doi: https://doi.org/10.64898/2026.07.21.739819; this version posted July 21, 2026. The copyright holder for this preprint (which was not certified by peer review) is the author/funder, who has granted bioRxiv a license to display the preprint in perpetuity. It is made available under a CC-BY 4.0 International license.

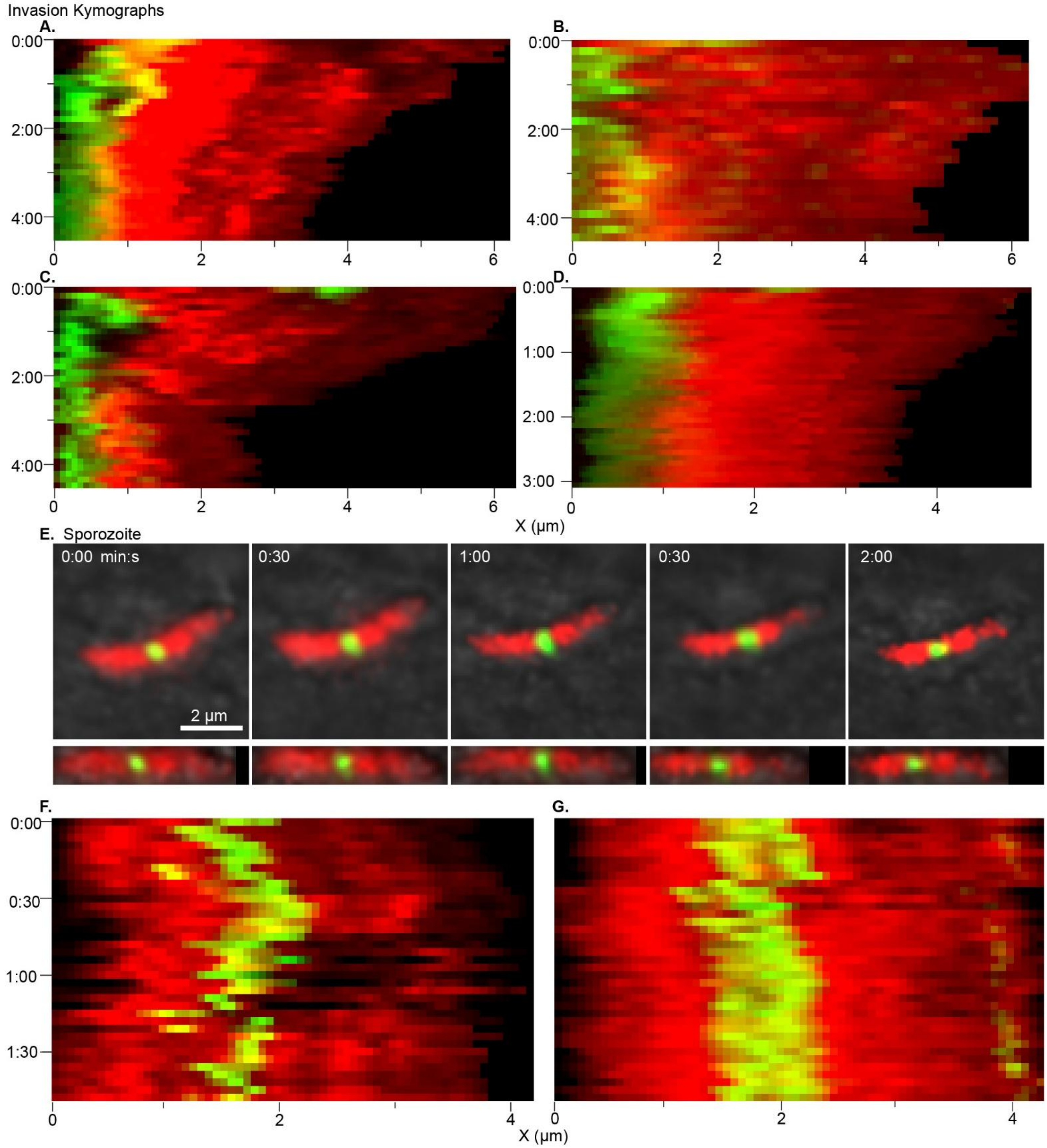

Figure S4. Additional time-lapse microscopy of PARG-mNeon secretion in invading and non-invading sporozoites. A-D. Additional kymographs showing the projected fluorescence intensity across the length of the parasite (X-axis) over time (Y-axis) from time-lapse microscopy of invading parasites showing changes in the distribution of PARG-mneon during invasion. The point of initial contact of the sporozoite with the HCT-8 cell is shown as time 0. **E.** Stills from a non-invading sporozoite imaged for 2 minutes. **F-G**. Kymographs showing the projected

bioRxiv preprint doi: https://doi.org/10.64898/2026.07.21.739819; this version posted July 21, 2026. The copyright holder for this preprint (which was not certified by peer review) is the author/funder, who has granted bioRxiv a license to display the preprint in perpetuity. It is made available under a CC-BY 4.0 International license.

fluorescence intensity across the length of the parasite for two examples of non-invading sporozoites.

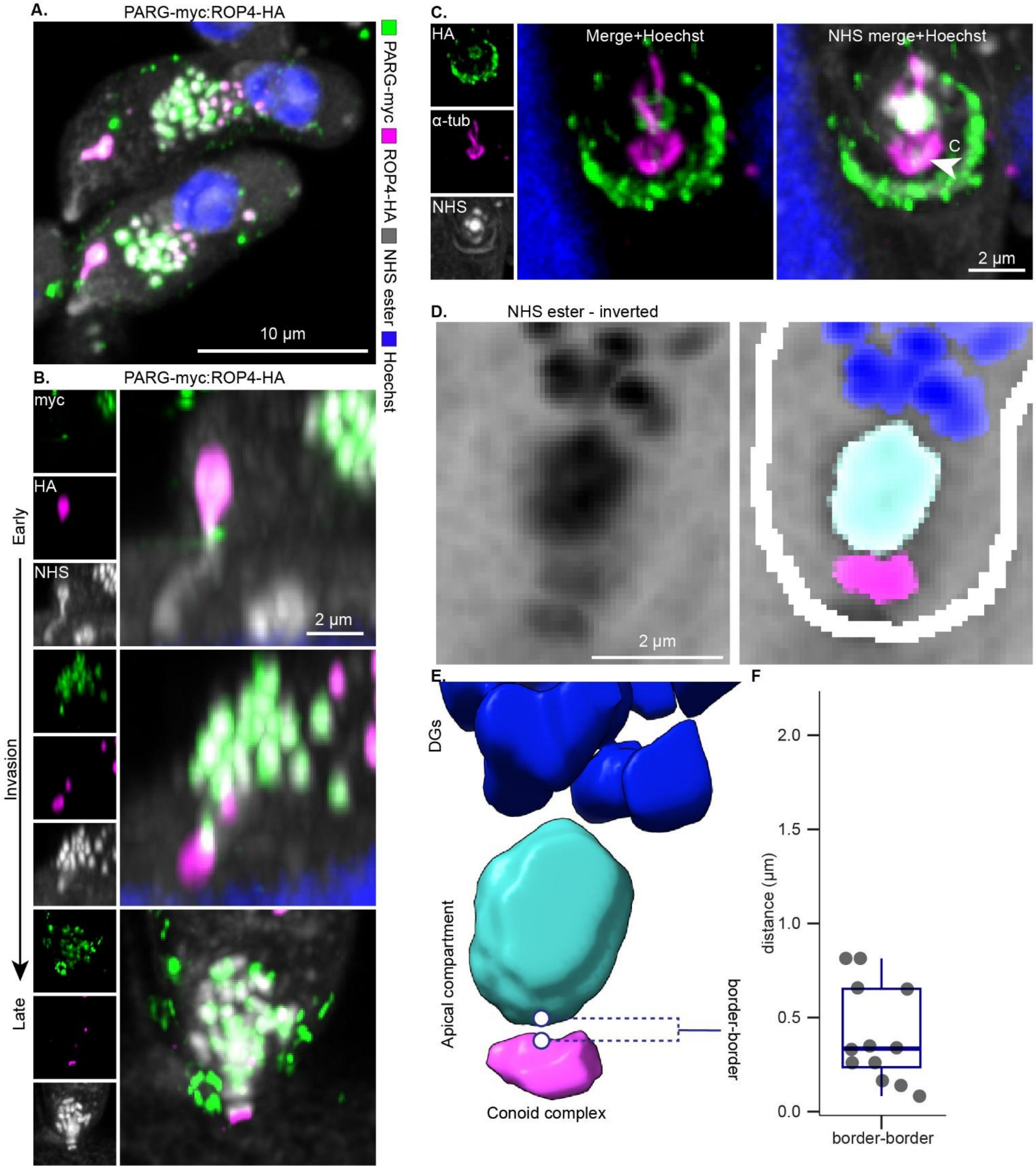

Figure S5. Rhoptry secretion precedes dense granule secretion, and the apical compartment is at a fixed position relative to the conoid complex. A-B. To simultaneously label the rhoptry and dense granule contents, PARG was myc-tagged at its endogenous location and Rop4-HA was knocked-in downstream with 500bp upstream of the start codon (PARG-

bioRxiv preprint doi: https://doi.org/10.64898/2026.07.21.739819; this version posted July 21, 2026. The copyright holder for this preprint (which was not certified by peer review) is the author/funder, who has granted bioRxiv a license to display the preprint in perpetuity. It is made available under a CC-BY 4.0 International license.

myc:ROP4-HA). Myc is shown in green, HA is in magenta, NHS ester is in gray and Hoechst is in blue. **A.** merged panel showing two sporozoites labelled with ROP4 in the rhoptry bulb and PARG in the dense granules. **B.** The apical end of sporozoites at different stages of invasion are shown. The first example shows the rhoptry tip inserted into the host cell prior to discharge. In the next two panels, the rhoptry is no longer visible and the ROP4 signal has moved beyond the apical compartment. PARG-positive dense granules are still within the parasite. In the middle panel, they are beginning to cluster around the discharged rhoptry. In the last panel, they have started to fuse to form the apical compartment**. C.** Expansion microscopy of invading sporozoites expressing PARG-HA (staining in green) and stained with NHS ester (gray) and an α-tubulin antibody (magenta). The conoid complex (C) apical to the sub-pellicular microtubules is labelled with a filled arrow. Individual channels are shown on the left and tubulin-PARG-HA merge without and with NHS-ester are enlarged shown on the right. **D-F.** The apical compartment and the conoid complex were segmented in 3D using protein density from NHS ester labelling. **D.** An example of an inverted NHS ester contrast and segmentation overlay of the apical end of a parasite with the apical compartment (cyan), conoid complex (magenta), granules (blue), and parasite outline (white) shown. **E.** Rendering of the segmentation map in 3D. The distance measurement reported in F is illustrated. **F.** The shortest distance between the conoid complex and apical compartment borders was quantified and plotted on the right for 12 parasites. The edge of the apical compartment is 0.4 ± 0.26 μm (90 ± 47 nm scaled) away from the edge of the conoid complex.

bioRxiv preprint doi: https://doi.org/10.64898/2026.07.21.739819; this version posted July 21, 2026. The copyright holder for this preprint (which was not certified by peer review) is the author/funder, who has granted bioRxiv a license to display the preprint in perpetuity. It is made available under a CC-BY 4.0 International license.

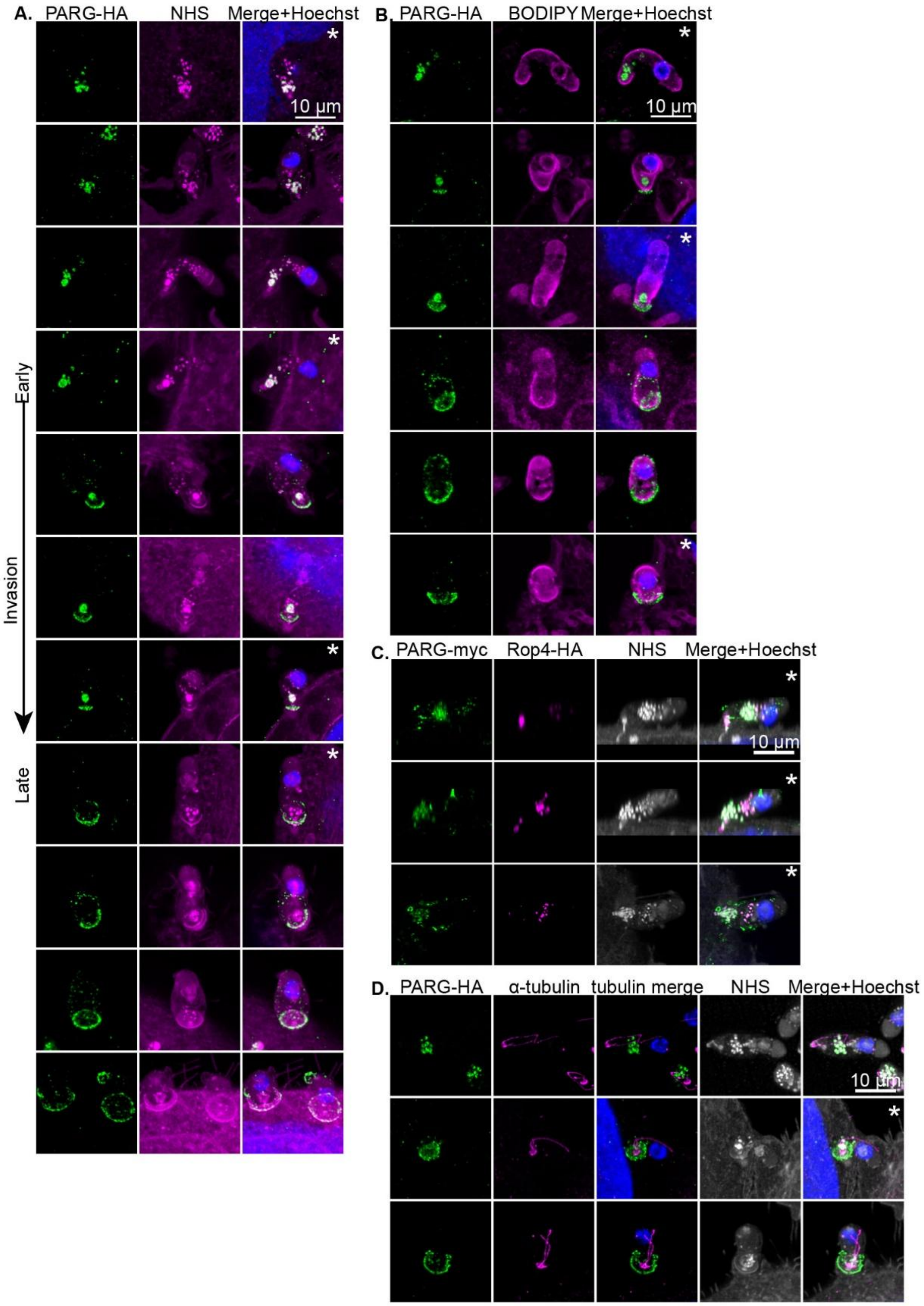

Fig. S6. Expansion microscopy atlas of *C. parvum* invasion. **A-D.** Additional examples of invading parasites stained with the following markers: **A.** PARG-HA + NHS ester, **B.** PARG-HA + BODIPY, **C.** PARG-myc:Rop4-HA and **D.** NHS ester, PARG-HA and tubulin. Images marked with an asterisk are whole parasite views that are cropped in Figs 4 and S5.

bioRxiv preprint doi: https://doi.org/10.64898/2026.07.21.739819; this version posted July 21, 2026. The copyright holder for this preprint (which was not certified by peer review) is the author/funder, who has granted bioRxiv a license to display the preprint in perpetuity. It is made available under a CC-BY 4.0 International license.

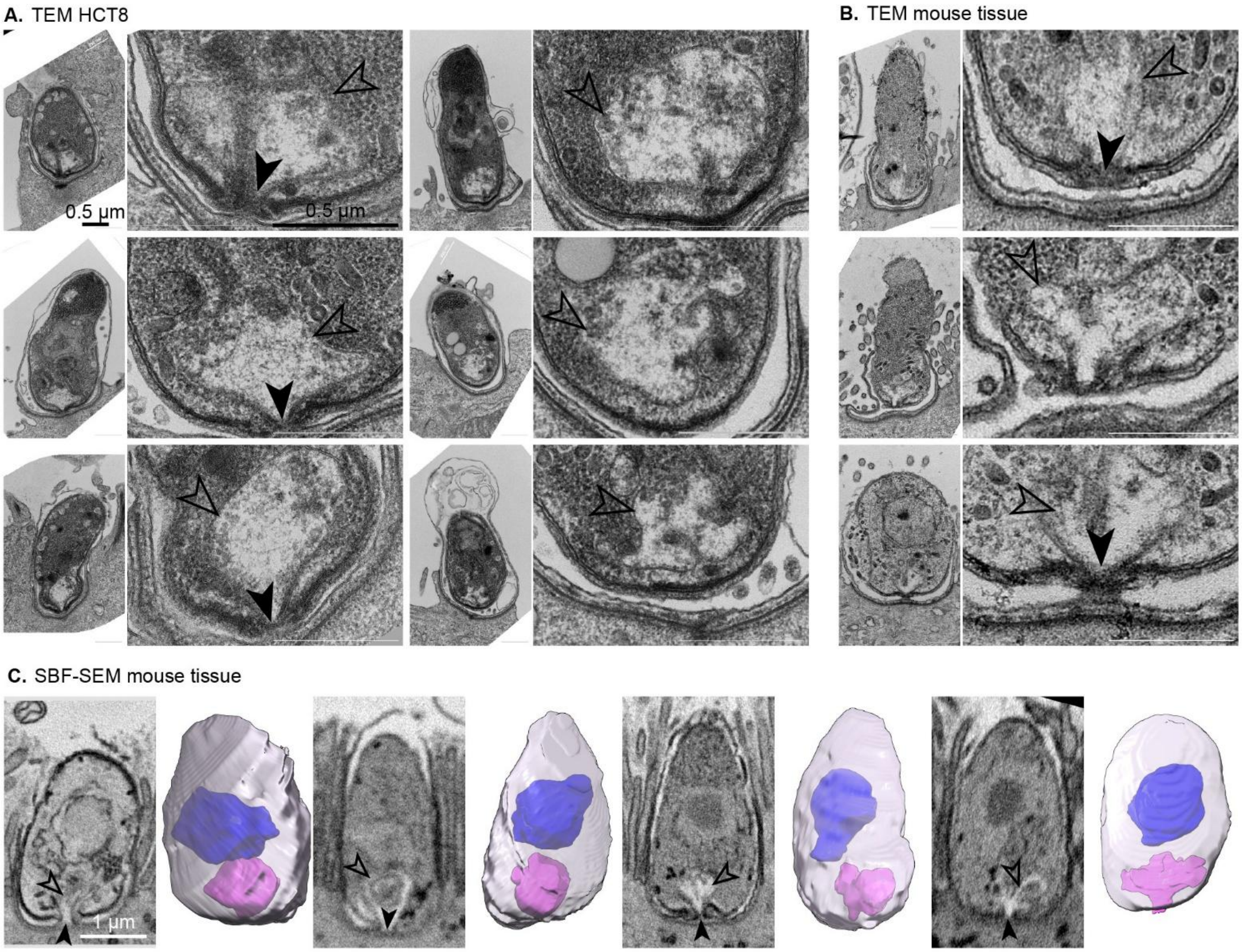

Fig. S7. Electron microscopy atlas of *C. parvum* invasion. Additional thin section TEM images of invading sporozoites and immature intracellular trophozoites interacting with HCT-8 cells (**A.**) and from infected mouse intestines (**B)**. Details on the right-hand side show the apical end. An electron translucent, membrane-bound compartment can be seen in all examples. **C.** Selected central slices and segmentation of the parasite membrane (gray), nucleus (blue) and apical compartment compartment (magenta) from serial block-face SEM imaging of intestinal sections from infected mice. Where visible, the boundary/membrane of the apical compartment is marked with an open arrow and the conduit connecting the apical compartment to the host cytosol is labelled with a filled arrow.

bioRxiv preprint doi: https://doi.org/10.64898/2026.07.21.739819; this version posted July 21, 2026. The copyright holder for this preprint (which was not certified by peer review) is the author/funder, who has granted bioRxiv a license to display the preprint in perpetuity. It is made available under a CC-BY 4.0 International license.

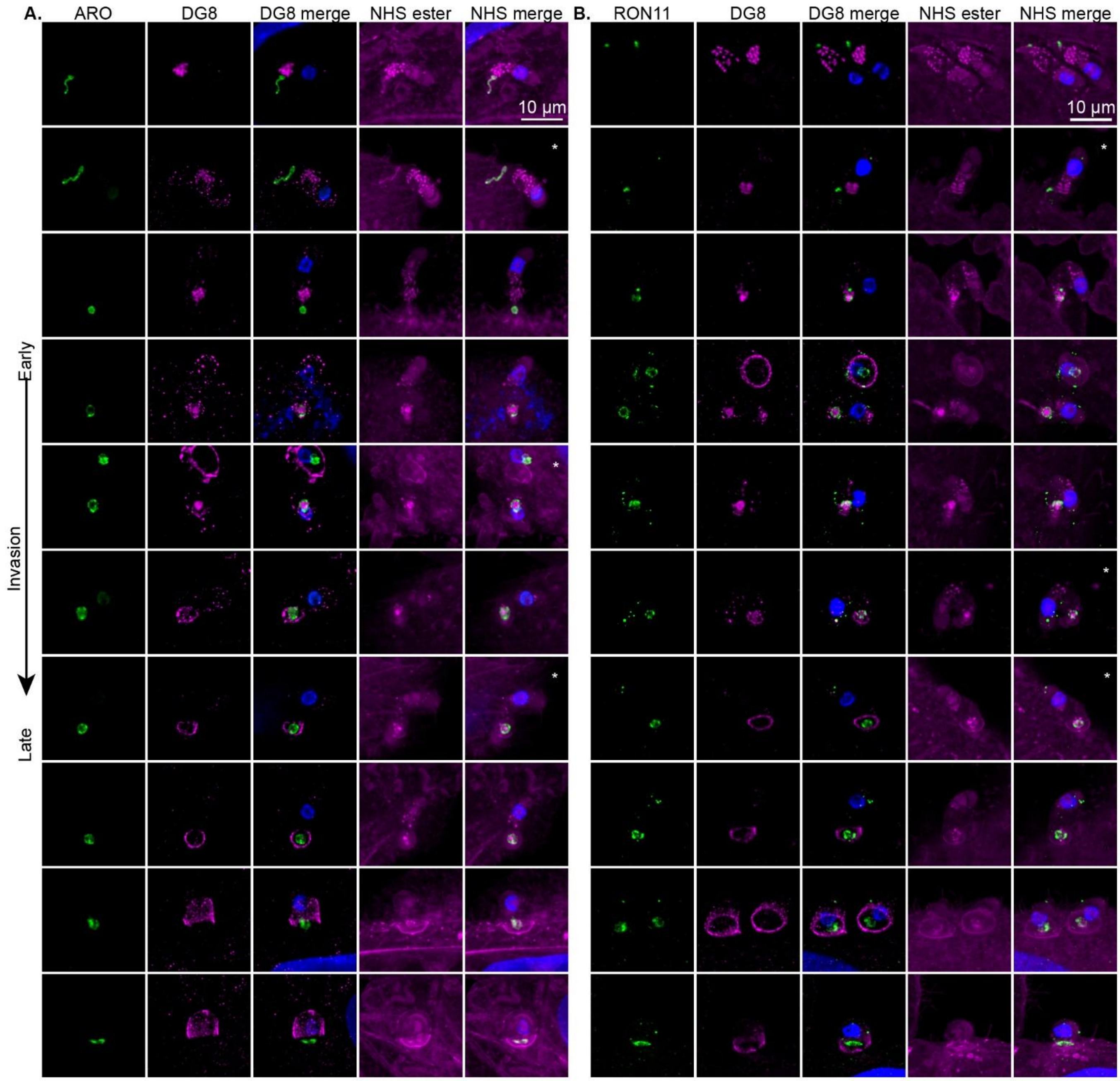

Figure S8. Expansion microscopy of rhoptry membrane markers during invasion. Additional examples of invading sporozoites and immature intracellular parasites visualized by expansion microscopy. Parasites expressed CpARO-HA (**A**) or CpRON11-HA (**B**). Immunofluorescence was used to detect HA epitopes (green) and DG8 (magenta). NHS ester was used to label protein density (Magenta) and Hoechst to label nuclei (blue). Images are arranged according to invasion stage from top to bottom. Images with an asterisk are full parasite views of cropped panels in Fig. 6.

bioRxiv preprint doi: https://doi.org/10.64898/2026.07.21.739819; this version posted July 21, 2026. The copyright holder for this preprint (which was not certified by peer review) is the author/funder, who has granted bioRxiv a license to display the preprint in perpetuity. It is made available under a CC-BY 4.0 International license.

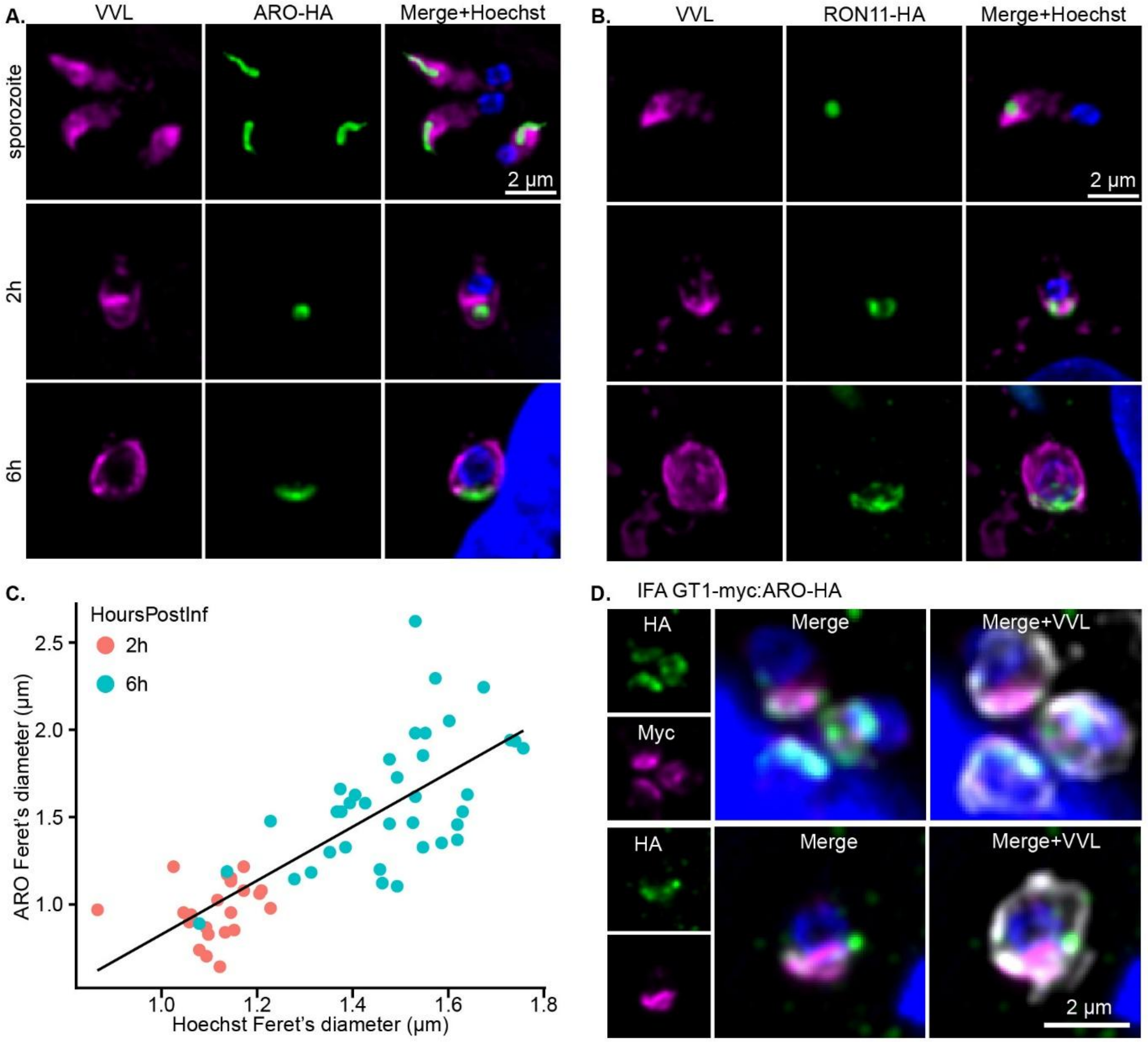

Figure S9. Immunofluorescence of CpARO and CpRON11 during trophozoite development. Representative examples of *C. parvum* parasites expressing **A.** CpARO-HA or **B.** CpRON11-HA at the sporozoite stage, the early trophozoite stage (2 hours post-infection) and the late trophozoite stage (6 hours post-infection. **C.** The size of the nucleus and ARO-HA-positive region were measured in maximum intensity projections. The maximum Feret's diameter of the nucleus (Hoechst) and the ARO-HA region are plotted. Points represent a single parasite, colored according to time point at which they were fixed (red = 2 hours post-infection, teal = 6 hours post-infection). Trendline: Pearson's Correlation Coefficient = 0.715, P-value = $1.38X10^{-10}$. Mean Feret's Diameter of ARO at 2h = 0.96 ± 0.16 µm, at 6h = 1.59 ± 0.37 µm. **E.** Immunofluorecence of GT1-myc and CpARO-HA localization in mature 1N meronts. HA is green, Myc is in Magenta, VVL is in gray, and Hoechst is in blue.

bioRxiv preprint doi: https://doi.org/10.64898/2026.07.21.739819; this version posted July 21, 2026. The copyright holder for this preprint (which was not certified by peer review) is the author/funder, who has granted bioRxiv a license to display the preprint in perpetuity. It is made available under a CC-BY 4.0 International license.

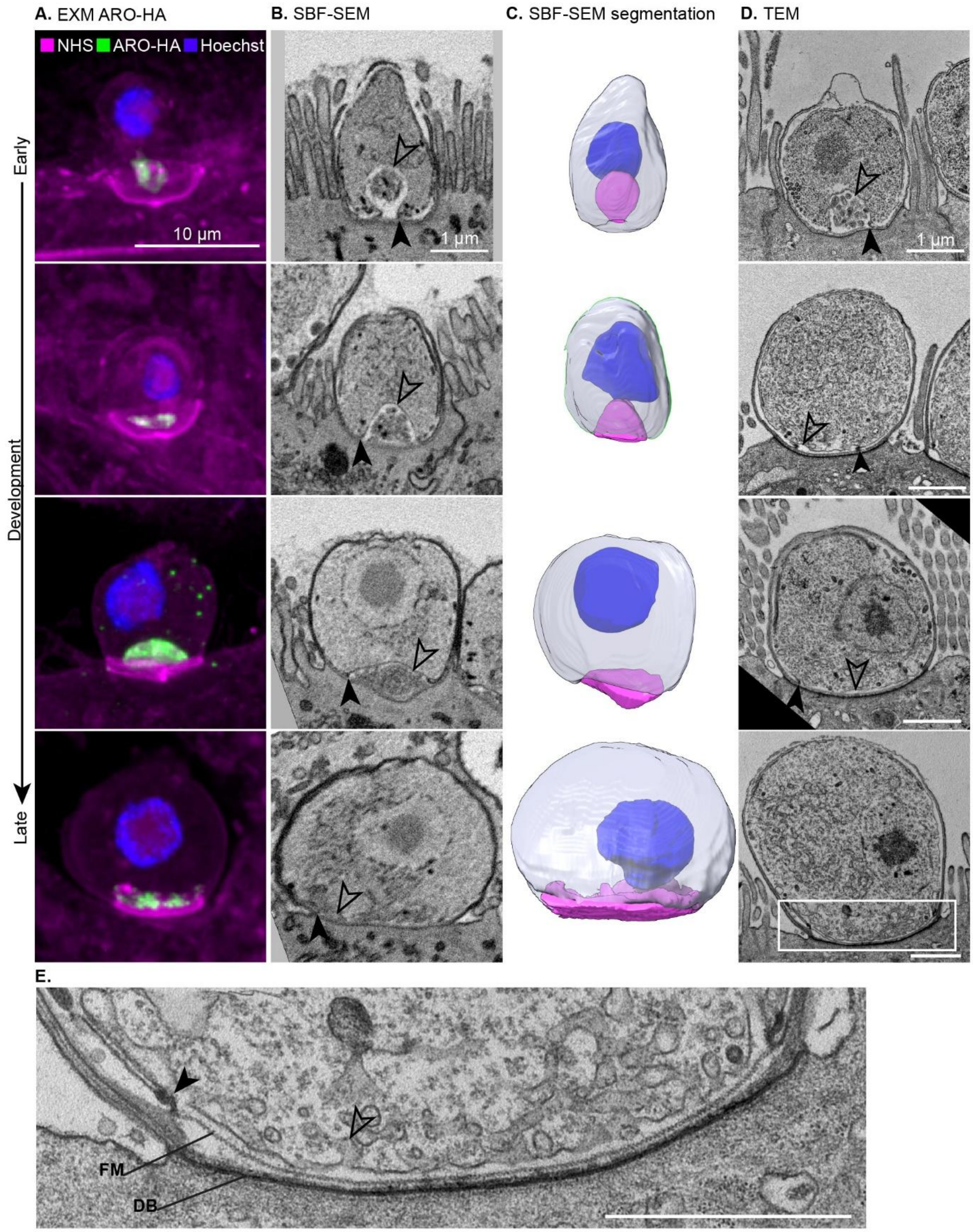

Figure S10. Comprehensive electron microscopy and expansion microscopy of feeder organelle development A. Expansion microscopy and ARO-HA staining at progressive stages of parasite development. **B.** Central slices from selected SBF-SEM volumes from infected mouse intestines showing the apical compartment transitioning into the feeder organelle (top to bottom). The boundary of the apical compartment is shown with an open arrow and the annular ring with a filled arrow. **C.** Segmentation of the nucleus (blue), apical compartment/feeder organelle

bioRxiv preprint doi: https://doi.org/10.64898/2026.07.21.739819; this version posted July 21, 2026. The copyright holder for this preprint (which was not certified by peer review) is the author/funder, who has granted bioRxiv a license to display the preprint in perpetuity. It is made available under a CC-BY 4.0 International license.

(magenta) and parasite boundary (transparent gray) and rendering in 3D. **D.** Thin section TEM from infected mouse intestines showing the apical compartment transitioning into the feeder organelle (top to bottom). The boundary of the apical compartment is shown with an open arrow and the annular ring with a filled arrow. **E.** Detail from thin section TEM (cropping area shown in the last image in panel **D)** showing the host-parasite interface of a mature intracellular parasite at high resolution. The dense band (DB), filamentous mesh (FM), feeder organelle membrane (open arrow) and annular ring junction (filled arrow) are labelled.

Supplementary Tables

Table S1. Oligonucleotides used in this study

Name	Sequence
AC001	GTTGGAGAGGACATTGAAAACGTTT
AC002	AAACAAACGTTTTCAATGTCCTCTC
AC003	AAGGGAACGCTCTCAAAGTGATGTTAATGggaagtggaggacgggaattc
AC004	CTAAAAAATATTAGCTTTGAAACACGTCCCcgcgtttaaactgattggtacta
AC005	AAGTTgtgtgaatgtcatctttagtG
AC006	AAAACactaaagatgacattcacacA
AC007	AGCATACACTCGAAGCGAACATC
AC008	GGAGGAGTTTTTGGGGGAGA
AC009	TCTAGCTGCGTATGCCGATG
AC011	GTGCTGACGGGACGCGCCGATATTCGGCCAatacgactcactataggg
AC012	ACCAGTTGTCAGCAGGTGAT
AC014	GTTCTGGCAGGCTACAGTGA
AC015	TTCCCAGCTGTGATTGCGTT
AC009	TCTAGCTGCGTATGCCGATG
AC016	gttggTGTGCCCGAAAAGAATTTAG
AC017	aaacCTAAATTCTTTTCGGGCACAc
AC018	TCAAGAATTGAATCTTCATTTTCAAGTGCATAAtggggaaactaaatatactg
AC019	GATAATACTGAAAATGTGCCCGAAAAGAATCGCGTTTAAACtgattggtACTA
AC020	AGTTTGTGAAACTCTGCTAGGAC
AC021	TCGTGTGAACATCTGTATTGAGT
AC027	CGCGTTTAAACTGATTGGTAC
AC028	TGAAAATGGTCGTTTTTCTGGTTTCATCGACTG
AC035	ACTGCCTTTCGCTTGGGATA
AC052	AAATGGTCGTTTTTCTGG
AC053	AATTAAGATAAAAAGAAAAACTTAATCG
AC046	TCGTTGTGGAAAGAGCTCCTCTACGGTGCAggctcttacccgtacg
AC047	AAGTTggactcccgtggaagaagatG
AC048	AAAACatcttcttccacgggagtccA
AC049	gaatttggacgctgaaactgtttgatcaagCAGCACGAAACCTTGCATTCA
AC050	ATAGTCGGAACGTGTGCTGTC
AC113	ggtagcggtggacgtgag

bioRxiv preprint doi: https://doi.org/10.64898/2026.07.21.739819; this version posted July 21, 2026. The copyright holder for this preprint (which was not certified by peer review) is the author/funder, who has granted bioRxiv a license to display the preprint in perpetuity. It is made available under a CC-BY 4.0 International license.

AC114	agtttttctttttatcttaattttttcagctaaattaacctctaatag
AC115	aactcacgtccaccgctaccaaaataagtattaacctcttgttc
AC152	gttggATATTAGGAACTTAAGGCTG
AC153	aaacCAGCCTTAAGTTCCTAATATc
AC154	GAGCTGGAGGAGGAAATTGTTGAGCGCATTggaagtggaggacgggaattc
AC155	AAATTAAACTAATCGGCTAAATAAATATAAcgcgtttaaactgattggtacta
AC172	CCATTGAAGGTGGAATCCCCA
AC173	TTAAGCCCCAATCAGCAACT
AC176	agtttttctttttatcttaattaagaatattttacctgcatgc
AC177	aactcacgtccaccgctaccctcagcaactctagctaataaaatttc
AC178	GAAGAAGATTCAGTTAAGAATCCAGATCTCggaagtggaggacgggaattc
AC179	CTTCTCCCTTCTTCTTCCTTTTAAGTCCCTcgcgtttaaactgattggtacta
AC180	GTTGGAAGGCCAACTTTGTAGAATA
AC181	AAACTATTCTACAAAGTTGGCCTTC
AC196	CCAGCTAGCTCTTTCACCAGT
AC197	AGCTCTGTAGAAACCAAGGCT
AG56	GTTGgaactaatgcttattctaaa
AG57	AAACtttagaataagcattagttc
AG95	gctgtcccgtgagatataga
AG97	TCGTATCGAACGTGCTCGTA
AG99	GGAATATACACGCGCTATTCG
AG100	gggaaatatcgatctattctg
AG339	TCGAGTGCCCAGGATTCATT

Table S2. Oligonucleotide pairs for guide RNA cloning and homology-directed repair template amplification.

Transgenic	GeneID(s)	Guide oligos	repair oligos
PARG-HA	Cgd8_2160	AC001/AC002	AC003/AC004
PARG-KO	Cgd8_2160	AC016/AC017	AC018/AC019
PARG-mNeon:tdTomato	Cgd8_2160	AC001/AC002	AC003/AC004
TgPARG2-HA	TgRH-280380	AC005/AC006	AC046/AC011
TgPARG2-KO	TgRH-280380	AC047/AC048	AC049/AC011
PARG-tdTomato	Cgd8_2160	AC001/AC002	AC003/AC004
PARG-myc:Rop4-HA	Cgd8_2160, Cgd3_1730	AC001/AC002	AC003/AC004
CpARO-HA	Cgd2_370	AG56/AG57	AC142/AC143
CpRON11-HA	Cgd3_2010	AC152/AC153	AC154/AC155
CpGT1-myc:ARO-HA	Cgd3_4070, Cgd2_370	AC180/AC181	AC178/AC179

Table S3. Oligonucleotide pairs for integration PCR and expected sizes

bioRxiv preprint doi: https://doi.org/10.64898/2026.07.21.739819; this version posted July 21, 2026. The copyright holder for this preprint (which was not certified by peer review) is the author/funder, who has granted bioRxiv a license to display the preprint in perpetuity. It is made available under a CC-BY 4.0 International license.

Transgenic	5'int (a/c)	expected size (bp)	3'int (b/d)	expected size (bp)	span (a/b)	expected size WT (bp)
PARG-HA	AC008/AG95	458	AC009/AG97	600	AC008/AC009	391
PARG-KO	AC020/AG95	332	AC021/AG97	588	AC020/AC021	423
PARG-mNeon:tdTomato	AC008/AC058	589	AC009/AC035	765	AC008/AC009	391
TgPARG2-HA	AC012/AC051	1748	AC014/AC015	488	AC012/AC015	549
TgPARG2-KO	AC050/AC051	512	AC014/AC015	488	AC050/AC015	2734
PARG-tdTomato	AC008/AG95	1796	AC009/AG97	600	AC008/AC009	391
PARG-myc:Rop4-HA	AC008/AG95	458	AC009/AG339	538	AC008/AC009	391
CpARO-HA	AG99/AG95	351	AG100/AG97	591	AG99/AG100	378
CpRON11-HA	AC172/AG95	481	AC173/AG97	689	AC172/AC173	513
CpGT1-myc:ARO-HA	AC196/AG95	531	AC197/AG99	495	AC196/AC197	488

Supplementary Movie Legends

Movie S1. PARG-mNeon:tdTomato invasion (related to Fig. 2, S4). Time lapse microscopy of 5 invasion events. Sporozoites express PARG-mNeon (green) to label the contents of dense granules and cytosolic tdTomato (red). Bright field/DIC is shown in gray. Invasion movies 1-4 were acquired using an OMX SR Delta Vision microscope. Invasion movie 5 was recorded using a CrestOptics X-light V3 Spinning Disk Confocal. Additional details are available in the Method Details section. Movies are cropped to show frames prior to significant photobleaching. Scale bar = 5 μm.

Movie S2. PARG-mNeon:tdTomato non-invading (related to Fig. 2, S4). Time lapse microscopy of 3 attached sporozoites that do not invade. Sporozoites express PARG-mNeon (green) and cytosolic tdTomato (red). Brightfield is in gray. Images were acquired using an OMX SR Delta Vision microscope. Scale bar = 5 μm.

Movie S3. PARG-tdTomato on Lifeact-GFP HCT-8 cells invasion (related to Fig. 3A-B). Time lapse microscopy of a sporozoite expressing PARG-tdT (green) invading an HCT-8 cell expressing GFP-lifeact (red). Brightfield is in gray. Images were acquired using an OMX SR Delta Vision microscope. Scale bar = 2 μm.

Movie S4. PARG-tdTomato on PLC-δ-GFP HCT-8 cells invasion (related to Fig. 3C-D) Time lapse microscopy of a 2 invasion events. Sporozoites express PARG-tdT (green), and HCT-8 cell expressing GFP-PLC-δ (red). Brightfield is in gray. Image 1 was acquired using an OMX SR Delta Vision microscope. Image 2 was acquired using a CrestOptics X-light V3 Spinning Disk Confocal. The bright-field channel is not shown in Image 2 due to focus issues. Scale bar = 2 μm.

Movie S5. Segmentation and rendering of expansion microscopy volume (related to Fig. 4, S5). Animation through a Z stack of an invading parasite expressing PARG-HA imaged by expansion microscopy. PARG-HA is in green, NHS ester is in magenta and Hoechst is in blue. Amination through the Z stack showing just NHS ester displayed with inverted contrast (black on white). Segmentation of key features (parasite = gray, conoid complex = magenta, granules = blue, apical compartment = cyan) is overlayed in the next stack, and the 3D rendering is shown last. Scale bar = 10 μm.

bioRxiv preprint doi: https://doi.org/10.64898/2026.07.21.739819; this version posted July 21, 2026. The copyright holder for this preprint (which was not certified by peer review) is the author/funder, who has granted bioRxiv a license to display the preprint in perpetuity. It is made available under a CC-BY 4.0 International license.

Movie S6. SBF-SEM volume of invading parasites, segmentation, and rendering (related to fig. 5C). Amination through 5 SBF-SEM Z-stacks of invading (Volume 1) and early-stage intracellular (Volume 2-5) *C. parvum* parasites in mouse intestinal tissue. Segmentation and rendering of the apical compartment (magenta), parasite (gray) and nucleus (blue) are shown. Scale bar = 0.5 µm.

Movie S7. TEM tomography of invading parasites (related to fig. 5E). Amination through a tomogram in Z reconstructed from a tilt-series acquired from a *C. parvum* sporozoite in the process of invading an HCT-8 cell *in vitro*. Scale bar = 0.5 µm.

Movie S8. SBF-SEM volume and segmentation of immature, maturing, and immature intracellular parasites (related to Fig. S10). Animation through 4 SBF-SEM Z stacks of parasite in mouse intestinal tissue with rendering of the apical compartment/feeder (magenta), parasite (gray), and nucleus (blue) segmentations. Volumes illustrate progressive development of the apical compartment into the feeder organelle as intracellular parasites mature. Scale bar = 1 µm.
